# Academic health centers: integration of clinical research with healthcare and education. Comments on a workshop

**DOI:** 10.6061/clinics/2017/e515s

**Published:** 2018-09-11

**Authors:** Roberto Jun Arai, Irene de Lourdes Noronha, José Carlos Nicolau, Charles Schmidt, Gustavo Moreira de Albuquerque, Kenneth W Mahaffey, Eduardo Moacyr Krieger, José Otávio Costa Auler Júnior

**Affiliations:** I Instituto do Cancer do Estado de São Paulo (ICESP), Hospital das Clinicas HCFMUSP, Faculdade de Medicina, Universidade de Sao Paulo, Sao Paulo, SP, BR; II Comissão de Facilitação de Pesquisa Clínica, Hospital das Clinicas HCFMUSP, Faculdade de Medicina, Universidade de Sao Paulo, Sao Paulo, SP, BR; III Laboratório de Nefrologia Celular, Genética e Molecular, Faculdade de Medicina, Universidade de Sao Paulo, Sao Paulo, SP, BR; IV Instituto Central, Hospital das Clinicas HCFMUSP, Faculdade de Medicina, Universidade de São Paulo, Sao Paulo, SP, BR; V Stanford Center for Clinical Research, Stanford University, California, USA; VI Instituto do Coração, Hospital das Clinicas HCFMUSP, Faculdade de Medicina, Universidade de Sao Paulo, Sao Paulo, SP, BR; VII Faculdade de Medicina, Universidade de Sao Paulo, Sao Paulo, SP, BR

Formal training to prepare physicians for careers as investigators in Brazil is still incipient 1. However, the learning environment is changing dramatically. The competitive arena is transforming the traditional learning environment into a collaborative and flexible learning environment 2. Clinical researchers and practitioners ideally share a goal of increasing the interactions between research and routine practice in an evidence-based medicine (EBM) environment. EBM frameworks promote clinically relevant research and consequently shape the scientific nature of the trainee's professional development. Early experience in clinical research represents a powerful opportunity to train medical students to recognize the importance of training with new technologies. Those students are likely to adopt and maintain this approach as they progress through further professional achievements.

The Academic Health Centers (AHCs) define a mission that engages education, research and advances in clinical care. Determining the value of its integration requires consideration of all stakeholders involved in the support for, investment in and profit from a center. The strategic planning for each institution can be implemented if leadership understands the importance of its internal stakeholders' contributions and how to create value from integration. Institutions should identify strategies to successfully create value from a collaborative engagement that cannot be achieved if the three elements operate alone. The concept of integration should be improved to become one of the key elements of institutional culture. Groups and departments that enhance integration of the three elements of AHCs should be recognized and rewarded to strengthen their integrative culture.

The workshop was organized to bring together leading experts in the field to comprehensively analyze the current state and future developments of the research, education and healthcare environment in the Hospital das Clínicas da Faculdade de Medicina da Universidade de São Paulo, Brazil (HCFMUSP). The workshop took place on September 14^th^, 2017, in São Paulo, Brazil, and included more than 30 members of the Hospital das Clínicas and three international representatives from Charité University, Stanford University, and the University of Coimbra. Each international representative provided a presentation with his/her experience. After the presentations, the working groups included invited members for discussions.

The discussing group of clinical research topics addressed critical points in integrating the human research program to education and healthcare. The information raised was organized and considered useful in providing insights and recommendations. The content of this manuscript was prepared by the members of the clinical research committees. The manuscript attempts to summarize the most salient points from members' reports and to provide the framework for a coordinated effort to address each of the discussed topics.

The HCFMUSP is a hospital complex located in the city of São Paulo, Brazil. The HCFMUSP is an autarchy of the State of São Paulo and is associated with the Faculty of Medicine of the University of São Paulo (FMUSP). The mission of the HCFMUSP is to provide medical education and research in order to deliver excellency in health services provided to the community.

The healthcare at the HCFMUSP is concentrated on disease prevention, medical-hospital care, tertiary care, and rehabilitation. Academically, the HCFMUSP provides undergraduate courses and broad and specific postgraduate courses. Research in most of the branches of the health sciences is conducted through its 62 medical research laboratories and clinical research centers in specialized institutions 3,4.

Since its inauguration on April 19, 1944, the HCFMUSP has been considered one of the most important Brazilian AHCs for the dissemination of technical and scientific knowledge 4. The HCFMUSP has approximately 380,000 m^2^, with >2,000 beds and >15,000 professionals in a variety of fields and specialties in different facilities as follows:

- Central Institute or *Instituto Central (ICHC)*
- Ambulatory Building or *Prédio dos Ambulatórios (PAMB)*
- Orthopedic and Trauma Institute or *Instituto de Ortopedia e Traumatologia (IOT)*
- Psychiatry Institute or *Instituto de Psiquiatria (IPq)*
- Child Institute or *Instituto da Criança (ICr)*
- Cancer Institute of the State of São Paulo or *Instituto do Câncer do Estado de São Paulo (ICESP)*
- Heart Institute or *Instituto do Coração (InCor)*
- Radiology Institute or *Instituto de Radiologia (InRad)*
- Rehabilitation and Physical Medicine Institute or *Instituto de Medicina Física e Reabilitação (IMREA)*
- Auxiliary Hospital of Suzano or *Hospital Auxiliar de Suzano (HAS)*
- Auxiliary hospital of Cotoxo or *Hospital Auxiliar de Cotoxó (HAC)*
- Units
- Administration Building or *Prédio da Administração*
- AIDS House or *Casa da AIDS*
- Rebouças Convention Center or *Centro de Convenções Rebouças (CCR)*
- Laboratories
- Medical Research Laboratories or *Laboratório de Investigações Médicas (LIM) 1 to 62.*

## THE IMPORTANCE OF AN INTERNATIONAL NETWORK

With the emerging global burden of chronic diseases, infections and inequalities, it is crucial for AHCs to have a global reputation. Networking with international collaborators enriches discussions for potential solutions because of the integration of multinational perspectives. AHCs have emerged across the globe with a variety of institutional configurations and strategic approaches. However, universal elements are present among all of them. The three invited institutions shared experiences on improving research capacity in line with healthcare and strategies to successfully advance the medical education. The University of Coimbra brought experiences accumulated since its foundation in 1290, whereas the University of São Paulo was founded in 1935. The working group and others 5,6 reported a growing trend of academic partnerships between U.S., Canadian, and European health science institutions and AHCs from low- and middle-income countries. Research has been identified as one of the main reasons of this global movement and will gain increasing importance. For example, the change in paradigm in 2003 by the Human Genome Project unfolded new strategies in precision clinical research. The use of patient molecular data to enrich participant selection for a given trial or a personalized treatment is becoming routine 7, although more slowly than initially forecasted. This new “taxonomy”, however, strongly limits accrual and, as a consequence, screening success has decreased drastically 8. To achieve sample targets, studies depend on multicenter and multinational collaborations to access a large, genetically diverse and pool of potential research participants 9,10. Precision clinical research is often complex and involves multiple stakeholders including physicians, the bioscience industry, high-tech laboratories, patients, and AHCs in several countries. These partnerships often encounter challenges such as resource disparities, which may affect expectations 5. The present workshop activities yielded an interactive process of action, assessment, and reflection to consider that project objectives and values should be aligned in order to ensure mutually beneficial objectives. In global diseases, multinational collaborations would produce powerful knowledge for humanity, as the involved AHCs worldwide would transfer or exchange technology and develop an updated research capacity for questions of the contemporary era.

## CLINICAL RESEARCH ENVIRONMENT

AHCs need to continuously update methodologies to support good research practices. Clinical research is conducted by principal investigators and/or departments in many Institutes of the HCFMUSP. The working group raised considerations about the lack of uniformity in the procedures and processes for research execution in Institutes housing several departments. One advantage of a decentralized model is to maintain research projects under the supervision of each department and/or the principal investigator, which often enhances faculty satisfaction based on greater sense of autonomy. Although this model appears valid, decentralized operations may result in duplicated infrastructure which underutilizes resources and requires distinct administrative approaches to adapt to the culture of different departments. This model is also not scalable if institutions invest in clinical investigators and the volume of clinical research grows, especially if it grows quickly. Compliance oversight, often not a priority of individual investigators, is more effective with a specific internal policy 11.

Investigators from the HCFMUSP wish to increase participation in rapidly evolving multinational research projects. In the last few years, the importance of multinational clinical research participation gained increasing attention resulting in the creation of a Board for Clinical Trials Facilitation to deliver solutions to the Office of Clinical Trials of the HCFMUSP in order to improve clinical research capacity 12. In line with the HCFMUSP organizational movement, that Board raised a critical point: the centralized model for research activities should be applied within each institute. In recent years, the institutional office of clinical trials (OCT) has emerged in AHCs to consolidate administrative activities related to clinical research 13. This organizational shift has typically increased research capabilities and management and has helped to achieve sustainability and growth. Similar to the University of Coimbra and the Stanford University Cancer Institute, the Cancer Institute, Heart Institute and Central Institute of the HCFMUSP have already established OCTs 14. Many interdependent functions are intersected with the research entity, which require an administrative structure for communications and interactions with distinct internal and external players. The emergence of OCTs is a demand of a growing requirement of stakeholders to be linked formally and harmonized in an efficient way 13. OCTs require institutional investment to support operational activities. Thus, the goals and results of OCT implementation should be clear to the leaders.

Clinical research activities are moving toward a high level of complexity in which investigators need support to accomplish all tasks. Some key activities of OCTs should include the following.

### Support for clinical trials agreements (CTAs)

Research projects often include sponsors or donations that will require institutional mediation or oversight. The formal legal instrument is a contract agreement between the parties. CTAs are separated from confidentiality agreements or investigators' agreements and are not regulated by regulatory agencies such as the Food and Drug Administration. CTAs are critical in dividing and protecting the responsibilities of each party, specifically the risks, financial support, rights and obligations. Usually, negotiation would need final approval of the legal department. Thus, the negotiators should be aware of basic legal requirements for clinical trial conduction. The costs of the institution, health insurance, clinical research insurance, percentage of overhead, hidden costs and other aspects of research budgeting should be considered and calculated as to avoid loses. Publication policy, intellectual and industrial property, data control and any other aspects of information should be stated in CTAs. A harmonized template containing all of the standard requirements of the institution and investigators should be adopted by OCTs.

### Clinical operations

Clinical trials require operational management to coordinate the project at any phase. The clinical trial coordinator at a local/institutional level will organize the logistic supply, treatment schedule, accrual activities and patient agendas. Protocol-related procedures are better controlled by a dedicated administrative trial coordinator from an OCT. Information technology and clinical trial management systems can be organized by the OCTs to provide best tools for each trial. One quality-related operation would be to conduct clinical trials in accordance with Good Clinical Practice (GCP) and International Conference of Harmonization (ICH) guidelines.

### Regulatory affairs

All clinical research is monitored by the government, as it is critical for safety reasons. Institutions would need a dedicated staff to liaise with regulatory bodies to avoid delays in receiving approvals. Regulatory liaison specialists can facilitate submissions to an institutional review board (IRB), an ethics committee (EC), and any other regulatory or sanitary authority. These specialists can organize the protocols' dossier and anticipate potential queries and pending documents. This group is critical to support investigators in maintaining good communication with regulatory bodies, which is a major aspect stated in the GCP guidelines.

### Training

GCP guidelines are a major standard for all clinical research. The ability to offer GCP training to all involved staff is desirable in order to harmonize data quality while strengthening the staff's ethical standards. Specific protocol-related training may also be organized by OCTs.

### Patient recruitment and retention

In the past decades, it has been difficult to achieve sample targets in clinical trials, while fewer patients are finishing studies 15. OCTs can provide support in maintaining an organized patient database to enhance accrual efficiency and help investigators with patient retention strategies.

### Quality oversight

Quality assurance of clinical research should be implemented to all research activities, particularly for safety reasons and for the quality of data. An organized OCT could implement or improve quality control by providing or promoting specific training and establishing staff minimum requirements for clinical research conduct. Each study may be monitored, and deviations or any other issues found in audit visits can be followed up. OCTs should organize a continuous process of quality improvements by revising communications and processes involving investigators and support staff.

### Encouraging and motivating physician-investigators

An important barrier that was noted during discussions in the workshop was the lack of time and motivation for research activities. Early career clinical investigators without proper training, dedicated research time or mentorship may experience substantial challenges and early failure in the conduct of clinical research, leading to a sense of emotional exhaustion. It is estimated that 16% of early career investigators experience burnout in this scenario. Moreover, a higher rate burnout (approximately one third) is found among physician-investigators who are over 35 years of age 16. Dedicated time for investigators to conduct research was a key consideration of the working group. Each institution would need to organize demands to allocate research activities while routine care is maintained (and improved). As per GCP guidelines, the investigator should have sufficient time to properly conduct and complete the trial within the agreed trial period (GCP 4.2.2) 17. It is also valuable for institutions to provide extra support to minimize personal hazards associated with professional burnout in all domains of the academic mission.

OCTs also act as supportive units for physicians to dedicate more time to clinical investigations. The academic physician-investigator faces several pitfalls. Obtaining funding to support research, more emphasis placed on clinical practice to generate funds, and increasing paperwork needed to comply with regulatory requirements are the tip of the iceberg 15,18. These facts may jeopardize the retention of young faculty members. OCTs may help in providing specialized support in navigating and coordinating clinical research ranging from the administration of contracts and budgets, subject recruitment and retention strategies, and data collection and quality to the care of research subjects 19,20.

### Compliance and integrity of AHCs

Recently, compliance activities have been critically important to ensure adherence to local, state and federal regulations pertaining to research, healthcare and education activities. Ties with pharmaceutical and device industries, among others, are common in medical research and practice, which may result in benefits through research collaborations that improve individual and public health. On the other hand, such relationships may create individual potential conflicts of interest. Such conflicts may threaten the integrity of scientific investigations, medical education and the quality of patient care 21. Research deviations, financial issues or any other misconduct behavior or process should be monitored by an organized commission that should work closely with the office of the general counsel and the office of internal auditing. Policies generally emphasize prevention and management rather than punishment. In AHCs, students may not understand the risks posed by conflicts of interests; thus, more education regarding compliance and integrity should be considered.

### Clinical research governance and the use of technology

The working group promptly indicated that the HCFMUSP is a complex organization. Multiple institutions, units and laboratories were built up on different occasions according to public healthcare demands of contemporary diseases. Leadership units are immediately facing internal structural challenges derived from faculty practices and distinct schools and cultures. Each institute poses particular governance characteristics. The working group has raised the importance of establishing strategic intersections in the centralized governance of the HCFMUSP, while maintaining the particularities of each institution.

The working group agreed that technology for healthcare and research purposes is constantly improving to enhance operational efficiencies. An integrated information system is necessary to link electronic medical records (EMRs) to decision support systems, supply control, work flows, and revenue cycle management programs to appropriately assessthe cost effectiveness of treatment, etc. An integrated system would necessitate the implementation of a management system covering the complete spectrum of care, from primary care units to specialized quaternary institutions. This system however, would need a policy for data control. Integrating any data from a myriad of distinct sources is a key solution to refine patient particularities but would create a tremendous volume of information under which conventional processing methods may simply collapse. Big data covers data retrieval, handling, and analyses of massive data 22. Integration of all data produced and managed by the HCFMUSP needs a careful review and analysis from the central governance perspective.

### Comprehensive cancer center

The working group considered the valuable, potential application of the Comprehensive Cancer Center model to the Cancer Institute of the HCFMUSP. The US National Cancer Institute (NCI) has characterized cancer centers based on their size and complexity. The largest type is the Comprehensive Cancer Center (CCC), which is devoted to clinical, basic and epidemiological cancer research to advance diagnostic, treatment and preventive methods in most or all cancers 23. CCCs can be organized as centers of excellence that are not limited to research but to patients looking for state-of-art treatment and access to clinical trials. The research is multidisciplinary, and complex studies are made possible by the collaboration and supportive technology platforms in the center structure. This would need a high capacity of articulating coworkers with common objectives. Thus, the organizational structure aims to bridge clinical programmers and supporting disciplines in a matrix to better achieve their interactions 23. The CCCs and cancer centers may be organized to collaborate in answering particular and urgent questions. In Brazil, the Ministry of Health and Ministry of Science, Technology and Innovation launched the National Clinical Trials Network (NCTN) in 2005, and a specific group was dedicated to oncology studies. This group aims to collaborate on projects that would not be feasible at single sites. The main objective of the NCTN is to promote health innovation facilitated by an organized infrastructure that eventually improves the national research capacity 24,25. The organization and modus operandi are, however, challenging and depend on the government initiatives. In 2009, the Cancer Institute of the HCFMUSP coordinated a NCTN multicenter study sponsored by the Brazilian National Council for Scientific and Technological Development (CNPq) 26. The project was implemented and successfully conducted (trial number NCT01370239). Nevertheless, the initiatives are still few. Recently, the reorganization of the NCTN was a topic of discussion in the meeting promoted by the Brazilian Ministry of Health to recover and to add greater strategic importance to the network 27.

The organization systems of CCCs may be applicable to Brazilian cancer centers. However, some critical points should be addressed. The internal organization of the cancer center should be multidisciplinary and often requires establishment or improvement of a collaborative culture. The OCTs may help in organizing CCCs, as this model gathers several departments and disciplines in response to common research questions. The CCCs and cancer centers are organized in a network for referral. For instance, many patients are referred to rehabilitation services of the University of Texas MD Anderson because is the only cancer center to have this facility 28. In Brazil, the policy for patient referral needs further improvement, as the demands for the public health system need to be better equalized.

Taken together, the group suggested recommendations (Table 1) to improve clinical research activities and strategies to better synchronize medical research with education and healthcare.

The role of clinical research in AHCs is expanding, as medical services face challenges imposed by new paradigms. Collaboration with different departments/institutes/units to integrate clinical trials with healthcare and education is always challenging, and all efforts should be continuously implemented to achieve the best possible level of organization. Here, we recommend some actions and suggestions for consideration. These suggestions should be adapted for each institution, as we understand that particular characteristics should be respected. It is noteworthy that the creativity of the investigator and inherent needs for independence and autonomy are important to preserve with the centralization of resources. Overall, the workshop was a great opportunity to exchange knowledge and experience to move forward in a complex scenario of clinical research of the modern era.

## AUTHOR CONTRIBUTIONS

Arai RJ, Nicolau JC and Mahaffey KW made substantial contributions to the conception of the manuscript. Noronha IL, Schmidt C and Albuquerque GM participated in drafting the article and revising it critically. Krieger EM and Auler Júnior JO organized the event, revised the article critically and give final approval of the manuscript. All authors participated in the discussions.

## Figures and Tables

**Table 1:** Recommendations for clinical research improvements and integration into medical education and healthcare in AHCs.

Incentive for networking	Develop strategies to improve international collaboration between AHCs to answer/overcome global challenges and exchange experiences
Promote research integrity	Adopt conflict of interest policies and integrity policies for AHCs and their institutions Implement and strengthen disclosure policies
Research culture	Implement or improve a comprehensive strategy to motivate and engage physician-investigators Develop a research culture together with the commitment to research quality, which are essential
Use of technology	Address information technology strains that are preventing data sharing and access among different providers Carefully consider the complex integration of data produced by the multi-institutional HCFMUSP from the central governance perspective
Office of Clinical Trials (OCT)	Consider developing OCTs to organize research activities under standardized policy; OCTs should consolidate critical research activities This would require institutional investment to support routine operational needs as follow OCTs would support the management of studies and regulatory submissions to the ethics committees and any other regulatory instances OCTs would oversee compliance enforcement, conduct regulatory and safety monitoring on an *ad hoc* basis and related to specific trials OCTs may offer support in using technology such as clinical trial management systems, electronic clinical report forms (eCRFs) and other tools related to clinical research databases, as well as data retrieval and analysis OCTs would support investigators with fiscal operations, increasing transparency for compliance purposes Trained staff to support clinical operations should be efficiently organized in terms of costs OCTs may offer support to small research groups or studies focusing on rare or neglected diseases
Governance	Establish an organizational plan updating the main priorities to deal with current and future research and healthcare challenges from the city, state and country perspectives Prioritize clearer values from collaboration among three elements: education, research, and healthcare Implement actions that reap the benefits from integration that otherwise would not be possible if operating alone Support new projects that promote the adequate use of resources such as equipment sharing and merging of replicated, specialized medical teams in corporate consolidated staff Consider the application and adaptation of the comprehensive cancer center model to specialized institute such as the Cancer Institute of HCFMUSP Consider the identity and culture of institutions as major aspects in the integration power of multi-institutional AHCs such as HCFMUSP

## References

[1] Coronel E, Fregni F (2015). Clinical research in Latin America: scientific production in high impact clinical research journals from 2000 to 2010. Int J Clin Trials.

[2] White LA, Krousel-Wood MA, Mather F (2001). Technology meets healthcare: distance learning and telehealth. Ochsner J.

[3] Economia (2009). Ranking de Clínicas y Hospitales de América Latina 2009 [Internet].

[4] Hospital das Clínicas da Faculdade de Medicina da Universidade de São Paulo (2018). HCFMUSP [homepage na internet].

[5] Luo A, Omollo KL (2013). Lessons learned about coordinating academic partnerships from an international network for health education. Acad Med.

[6] Mamzer MF, Duchange N, Darquy S, Marvanne P, Rambaud C, Marsico G (2017). Partnering with patients in translational oncology research: ethical approach. J Transl Med.

[7] Katsnelson A (2013). Momentum grows to make ‘personalized’ medicine more ‘precise’. Nat Med.

[8] Denicoff AM, McCaskill-Stevens W, Grubbs SS, Bruinooge SS, Comis RL, Devine P (2013). The National Cancer Institute-American Society of Clinical Oncology Cancer Trial Accrual Symposium: summary and recommendations. J Oncol Pract.

[9] DiMasi JA, Hansen RW, Grabowski HG (2003). The price of innovation: new estimates of drug development costs. J Health Econ.

[10] Arai RJ, Mano MS, de Castro G, Diz Mdel P, Hoff PM (2010). Building research capacity and clinical trials in developing countries. Lancet Oncol.

[11] Croghan IT, Viker SD, Limper AH, Evans TK, Cornell AR, Ebbert JO (2015). Developing a clinical trial unit to advance research in an academic institution. Contemp Clin Trials.

[12] Faculdade de Medicina da Universidade de São Paulo [homepage na internet] Escritório de Pesquisa Clínica HCFMUSP [access in 2018 mar 20].

[13] Rubin E, Lazar D (2009). Clinical Trials Offices: What’s New In Research Administration?. Issue Briefs Assoc Acad Heal Centers.

[14] ICESP [homepage na internet] (2018). Núcleo de Pesquisa.

[15] Malakoff D (2008). Clinical trials and tribulations. Spiraling costs threaten gridlock. Science.

[16] Primack BA, Dilmore TC, Switzer GE, Bryce CL, Seltzer DL, Li J (2010). Burnout among early career clinical investigators. Clin Transl Sci.

[17] ICH GCP [homepage na internet] Good Clinical Practice.

[18] Cadman EC (1994). The academic physician-investigator: a crisis not to be ignored. Ann Intern Med.

[19] Arai RJ, Longo ES, Sponton MH, Del Pilar Estevez Diz M (2017). Bringing a humanistic approach to cancer clinical trials. Ecancermedicalscience.

[20] Anderson C, Young PA, Berenbaum A (2011). Food and Drug Administration guidance: supervisory responsibilities of investigators. J Diabetes Sci Technol.

[21] Lo Be, Field MJ (2009). Institute of Medicine. Conflict of interest in medical research, education and practice.

[22] Raghupathi W, Raghupathi V (2014). Big data analytics in healthcare: promise and potential. Health Inf Sci Syst.

[23] Nathan D, Benz EJ (2001). Comprehensive cancer centres and the war on cancer. Nat Rev Cancer.

[24] Brasil Ministério da Saúde.

[25] De S, Insumos T (2010). Rede Nacional de Pesquisa Clínica do Brasil: respostas e redução da dependência estrangeira. Rev Saude Publica.

[26] Conselho Nacional de Desenvolvimento Científico e Tecnológico (CNPq) Edital 52/2009 [Internet].

[27] Brasil (2017). Ministério da Saúde. Evento de Ciência, Tecnologia e Inovação em Saúde 2017 [Internet].

[28] Shin KY, Guo Y, Konzen B, Fu J, Yadav R, Bruera E (2011). Inpatient cancer rehabilitation: the experience of a national comprehensive cancer center. Am J Phys Med Rehabil.

